# Regression Analysis of Combined Gene Expression Regulation in Acute Myeloid Leukemia

**DOI:** 10.1371/journal.pcbi.1003908

**Published:** 2014-10-23

**Authors:** Yue Li, Minggao Liang, Zhaolei Zhang

**Affiliations:** 1 Department of Computer Science, University of Toronto, Toronto, Canada; 2 The Donnelly Centre, University of Toronto, Toronto, Canada; 3 Department of Molecular Genetics, University of Toronto, Toronto, Canada; 4 Banting and Best Department of Medical Research, University of Toronto, Toronto, Canada; Memorial Sloan-Kettering Cancer Center, United States of America

## Abstract

Gene expression is a combinatorial function of genetic/epigenetic factors such as copy number variation (CNV), DNA methylation (DM), transcription factors (TF) occupancy, and microRNA (miRNA) post-transcriptional regulation. At the maturity of microarray/sequencing technologies, large amounts of data measuring the genome-wide signals of those factors became available from Encyclopedia of DNA Elements (ENCODE) and The Cancer Genome Atlas (TCGA). However, there is a lack of an integrative model to take full advantage of these rich yet heterogeneous data. To this end, we developed RACER (Regression Analysis of Combined Expression Regulation), which fits the mRNA expression as response using as explanatory variables, the TF data from ENCODE, and CNV, DM, miRNA expression signals from TCGA. Briefly, RACER first infers the sample-specific regulatory activities by TFs and miRNAs, which are then used as inputs to infer specific TF/miRNA-gene interactions. Such a two-stage regression framework circumvents a common difficulty in integrating ENCODE data measured in generic cell-line with the sample-specific TCGA measurements. As a case study, we integrated Acute Myeloid Leukemia (AML) data from TCGA and the related TF binding data measured in K562 from ENCODE. As a proof-of-concept, we first verified our model formalism by 10-fold cross-validation on predicting gene expression. We next evaluated RACER on recovering known regulatory interactions, and demonstrated its superior statistical power over existing methods in detecting known miRNA/TF targets. Additionally, we developed a feature selection procedure, which identified 18 regulators, whose activities clustered consistently with cytogenetic risk groups. One of the selected regulators is miR-548p, whose inferred targets were significantly enriched for leukemia-related pathway, implicating its novel role in AML pathogenesis. Moreover, survival analysis using the inferred activities identified C-Fos as a potential AML prognostic marker. Together, we provided a novel framework that successfully integrated the TCGA and ENCODE data in revealing AML-specific regulatory program at global level.

## Author Summary

Recent studies from The Cancer Genome Atlas (TCGA) showed that most Acute Myeloid Leukemia (AML) patients lack DNA mutations, which can potentially explain the tumorigenesis, and motivated a systematic approach to elucidate aberrant molecular signatures at the transcriptional and epigenetic levels. Using recently available data from two large consortia namely Encyclopedia of DNA Elements and TCGA, we developed a novel computational model to infer the regulatory activities of the expression regulators and their target genes in AML samples. Our analysis revealed 18 regulators whose dysregulation contributed significantly to explaining the global mRNA expression changes. Encouragingly, the inferred activities of these regulatory features followed a consistent pattern with cytogenetic phenotypes of the AML patients. Among these regulators, we identified microRNA hsa-miR-548p, whose regulatory relationships with leukemia-related genes including YY1 suggest its novel role in AML pathogenesis. Additionally, we discovered that the inferred activities of transcription factor C-Fos can be used as a prognostic marker to characterize survival rate of the AML patients. Together, we demonstrated an effective model that can integrate useful information from a large amount of heterogeneous data to dissect regulatory effects. Furthermore, the novel biological findings from this study may be constructive to future experimental research in AML.

## Introduction

One of the most intriguing questions in molecular biology is to decipher condition-specific transcription programs in complex organisms such as human [1]. The major transcriptional regulator proteins are transcription factors (TFs), which bind to cis-regulatory elements in the promoter regions of genes and control the downstream transcription activity [2]. MicroRNA (miRNA), a small 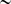
22 nucleotide noncoding RNA species, have been shown to play a predominant role in post-transcriptional and/or translational regulation [3]. While TFs can serve either as a transcriptional activator or repressor, miRNAs are primarily known to confer mRNA degradation and/or translational repression [4]. The coordinated transcriptional regulatory network comprising of miRNAs and/or TFs together maintains normal cellular state in a tissue-specific manner. Although abnormal miRNA and mRNA expression can be taken as strong indicator of carcinoma in clinical samples [5], [6], it is often unclear what causes the aberrant expression pattern and in particular how the transcriptional regulatory network differs between tumor and normal samples.

At the maturity of several high-throughput technologies, large-scale expression profiling of long and small RNAs by microarray or RNA-sequencing (RNA-seq) across hundreds of human samples as well as genome-wide interrogation of TF occupancy by chromatin immunoprecipitation sequencing (ChIP-seq) have become increasingly permissive. In particular, two major consortia namely Encyclopedia of DNA Elements (ENCODE) [7] and The Cancer Genome Atlas (TCGA) [8], [9] have made significant progress in generating and organizing the raw genomic data on public domains over the past few years. The primary goal of ENCODE is to decipher TF transcriptional regulatory network. To this end, the ENCODE team has generated high quality ChIP-seq data for over a hundred TFs in various human cell lines [7]. On the other hand, the TCGA consortium aims at identifying molecular signatures specific to each cancer type by profiling mRNA and miRNA expression as well as DNA methylation (DM), copy number variation (CNV) over hundreds of patient samples across many different cancer types.

With the availability of these huge amount of the data, there is an urgent need of developing computational models to effectively integrate heterogeneous data from multiple platforms and provide insightful directions for further focused research. Using ENCODE ChIP-seq data, [10] explored the topology of TF-miRNA coregulatory network by re-arranging the network into three layers based on TF-TF regulatory relationship, and then superimposed miRNA regulatory network derived from sequence-based predictions on top of the TF network. In a more recent work, [11] employed existing supervised machine-learning methods to examine how well the TF-binding signals can be used as predictors to explain the mRNA expression levels. These approaches achieved promising results: about 50% of the expression variances can be explained by TF binding data alone. However, the model prediction may be further improved by taking into account not only TF but also miRNA regulations.

Recently, [12] developed a regularized linear regression model in a glioblastoma (GBM) study using data from TCGA. The model was fit on mRNA expression changes in GBM tumor samples as the response variable using a linear combination of the input variables including the CNV, DM, miRNA sequence-based predictions (or miRNA expression), and binary TF-binding sites from TRANSFAC filtered by DNA hypersensitive regions from the ENCODE data. Despite the additive regulatory assumption, the model successfully identified various GBM-related regulators, whose estimated activities were predictive to GBM subtypes and patient survival rate. Their work demonstrated a parsimonious way to extract the most prominent TF/miRNA expression regulators by systematically dissecting different contributors, taking into account the confounding effects from CNV and/or DM. On the other hand, the model did not incorporate the valuable non-binary TF binding data from ENCODE. More recently, [13] proposed a similar regression framework to successfully predict 143 recurrent miRNA-mRNA interactions across 11 cancer types using the TCGA data. Their model, however, did not include TF regulations. Since TF are presumably the predominant expression regulators and miRNAs are known to primarily fine-tune transcriptional products [3], a model without considering TF regulations may be prone to overestimating the influences of the miRNAs.

In this study, we propose a novel two-stage regression framework that integrates data from both TCGA and ENCODE. The novelty of our model, named RACER (Regression Analysis of Combined Expression Regulation), lies in its ability to infer sample-specific TF activities using ENCODE TF binding data derived from a generic cell-line and then using the estimated regulatory activities to infer miRNA/TF-gene regulatory relationships across samples. As a case study, we choose Acute Myeloid Leukemia (AML) for the following reasons. Various high quality genome-wide measurements (CNV, DM, mRNA/miRNA expression measured by long/small RNA-seq) of a large cohort of 173 AML patients have very recently become available from TCGA (2013), providing a unique opportunity to study one of the most prevalent cancers [9]. Despite decades of efforts, the molecular pathogenesis of AML remains unclear. In particular, recent studies showed that, in contrast to many other cancers, the genomes of AML patients have fewer mutations or structural variation with an average of only 13 genetic mutations per patient, and that most of the recently predicted AML-related driver genes do not carry any mutations in the AML patients [9]. This motivates a novel way to interrogate AML cancer biology at the transcriptional and epigenetic level. Moreover, the AML samples are perhaps the best match to ENCODE Tier 1 K562 erythroleukemia cells (derived from a 53 year-old Chronic Myelogenous Leukemia patient), which by far has the highest number of 97 TFs measured by ChIP-seq experiments [14] and thus justifies our use of the TF occupancy in K562 as surrogates for the AML samples. By cross-validation, we show that our full regression model using various expression regulators indeed performs significantly better in predicting the held-out gene expression than the alternative models, where only a subset of the regulators are used as the input variables. Moreover, the proposed model demonstrates promising statistical power in detecting known or confidence TF/miRNA-gene interactions. Based on a feature selection procedure, we identified 18 prominent (post-)transcriptional regulators including 16 TFs and 2 miRNAs, whose inferred activities consistently cluster based on cytogenetic risk groups. Among the 18 selected regulators, we propose a novel role of a recently identified miRNA hsa-miR-548p in AML pathogenesis because of its significant target gene enrichments for leukemia-related pathway and its inferred interaction with another prominent feature regulator YY1, whose perturbed expression has been implicated in AML development through interference with the myeloid differentiation program in leukemic progenitor cells [15]. Moreover, we identified a potential prognostic marker using the inferred TF activities of C-Fos, which has been previously shown to have oncogenic activity and is frequently overexpressed in tumour cells [16].

## Results

### RACER: Regression analysis of combined expression regulation

**Figure 1:**
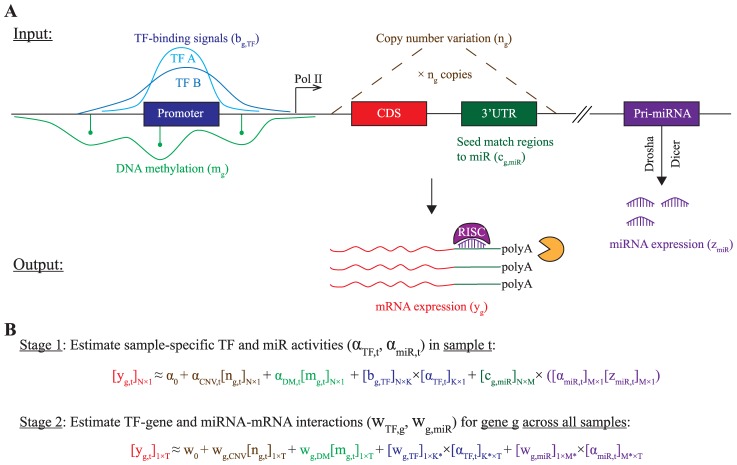
RACER schematics. **A**. The mRNA expression of gene 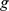 (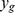) is modelled as a function of the following input variables (left to right): TF-binding signals (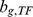), DNA methylation (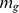), copy number variation (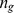), miRNA-mRNA interactions implicated in the sequence-based seed match (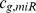) 3
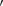
UTR regions of the mRNA and miRNA expression (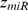). **B**. Two-stage regression analysis. At stage 1, RACER estimates the sample-specific TF and miRNA activities (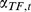, 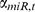) for each sample 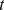. At stage 2, RACER uses the inferred regulatory activities of TFs and miRNAs to estimate the interaction scores 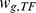 and 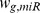 between gene 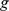 and TF and between gene 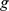 and miRNA 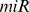 across all of the 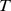 samples, respectively.

Changes in global gene expression profile may be the result of perturbation on distinct regulators, which may be specific even between subtypes of a broadly defined disease category. The objective of our work is to infer the predominant regulators of genes expression attributable to acute myeloid leukemia (AML) and their cognate mRNA targets. To this end, we took a reverse engineering approach in attempt to explain the gene expression by integrating transcriptional regulators, whose genome-wide measurements have recently become available from TCGA [9] or ENCODE [14]. In particular, we assumed that the changes of mRNA abundance are due to (1) copy number variation of the genomic DNA; (2) alteration of DNA methylation; (3) occupancy of transcription factors; (4) miRNA-mRNA interaction at the post-transcriptional level (Figure 1A). By dissecting the influence of these input variables in a regularized linear regression model (Figure 1B), we aimed to elucidate the hidden activities of the predominant TF and miRNA regulators and their target genes in AML. The idea of inferring TF/miRNA activities using linear models and subsequently inferring regulator-target connectivity are previously established in the literature [17]–[20], but (to the best of our knowledge) has not been applied to our problem formalism.

Specifically, we propose RACER (Regression Analysis of Combined Expression Regulation) that is specifically designed to integrate the above-mentioned explanatory variables to tackle this problem. Suppose there are 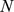 genes (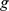), 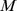 miRNAs (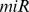), and 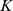 TFs. For each sample 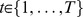, we observe expression levels of gene 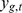 and miRNA 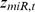 as well as CNV 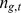 and DM signals 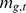 (together provided by TCGA [9]). The TF-binding signals, however, were not available for each sample but rather measured by ChIP-seq in a related cell line K562 (erythroleukemia cells; provided by ENCODE [14]). To integrate the sample-specific data from TCGA with the cell-line-derived TF-binding data from ENCODE, we devised a two-stage regression analysis. In the first stage, we estimate *sample-specific* TF and miRNA activities (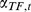, 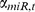) in sample 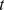 with 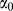 being the intercept, and 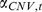 and 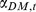 being the respective offsets for CNV and DM:
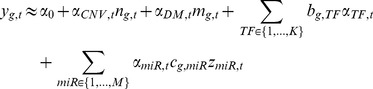
(1)where 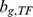 is the binding score of 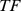 on gene 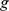, 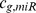 is the number of conserved target sites on the 3
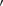
UTR of the target gene 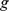 for 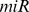, which is obtained as sequence-based information from TargetScan [21]. In the second stage, using the estimated 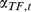 and 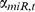 in (1), we infer for each gene 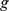 its association with the candidate TF (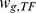) and miR regulators (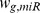) *across all of the* 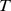 *samples*:
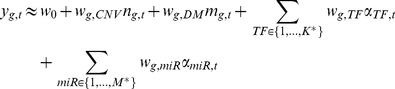
(2)where 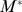 and 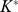 are the respective number of selected TFs and miRNAs with nonzero binding signals 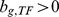 and conserved target sites 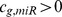 for gene 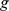. Notably, 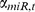 carries more dynamic information due to its association with the sample-specific miRNA expression levels 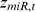 in (1). To obtain a robust estimate, we further weighted the 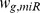 by the averaged activities of the miRNA: 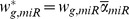, where 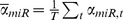. At each stage of RACER, we obtained a sparse LASSO solution [22], which minimizes the sum of squared errors with 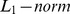 penalty on the linear coefficients (**Materials and Methods**). As reference, the following matrices were preserved for further analyses:

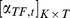
, 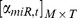: Activities of 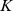 TFs and 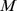 miRNAs across 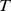 samples;

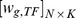
, 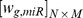: Predicted scores of regulatory relationships between 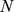 genes and 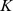 TFs or 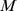 miRNAs.

In the AML data, 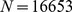, 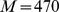, 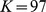, and 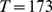 (Despite 200 samples reported in [9], we found 173 samples each having all of the above-mentioned data available).

### Comparison of the full and reduced/alternative RACER models

**Figure 2:**
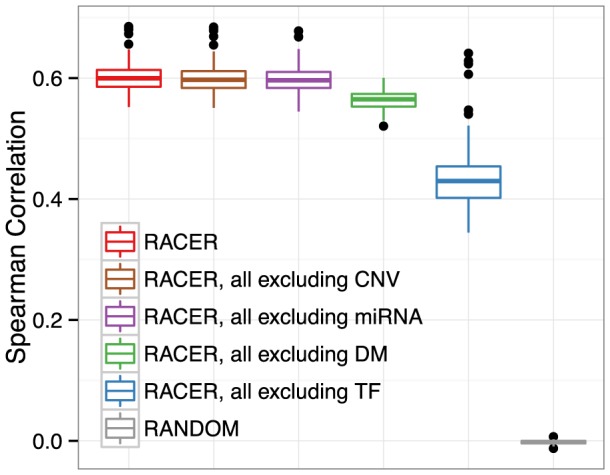
Model comparison. Boxplot of Spearman rank correlation between the predicted and the actual held-out genes in 10-fold cross-validation (CV). The full RACER model (red, left most) using all of the input variables was compared with the reduced model using all except for one type of the input variables “RACER, all excluding X”, where × is one of the following factors: CNV (copy number variation), miRNA (miRNA expression 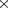 number of conserved target sites), DM (DNA methylation), TF (TF binding scores from ENCODE). RANDOM represents predictions from the complete RACER model on the same data but with gene labels randomly shuffled. The 10-fold CV was performed for each sample, and the average was taken over the 10 correlations. Each boxplot displays the distribution of the averaged correlation across the 173 AML samples. The higher the reduction relative to the full RACER model, the more power provided by the excluded variables in terms of explaining the underlying mRNA expression level.

To justify our model formalism, we compared the full RACER model with the models excluding one of the variables in terms of their abilities to explain expression changes in AML. Specifically, we performed a 10-fold cross-validation (CV) by training the full or reduced models on 90% of the genes and tested their predictions on the held-out 10%. The CV was carried out per sample-basis. After each CV run, we obtained the Spearman rank correlation [12] and coefficient of determination (
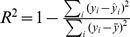
) [11] between the predicted and observed gene expression. Figure 2 (Figure S1) illustrates the CV results as a distribution of the correlation calculated across all samples for each model, which were ordered based on their median Spearman correlation (coefficient of determination). Encouragingly, the full RACER model performed the best and significantly better (p
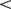
[1E-55, 0.2]; Wilcoxon signed rank test) than all of the other alternatives, achieving median Spearman correlation above 60% (Table 1). Because overfitting due to higher model complexity will result in poor CV performance, we attributed the superior performance of the full RACER model to its ability to extract unique biologically meaningful information from each type of input variable. Comparing the full with the partial RACER models (where one regulatory factor was excluded), it is remarkable to observe that the highest reduction of 20% is attributable to TF regulation followed by a 5% reduction when DNA methylation was omitted. In contrast, miRNA and CNV contributed modestly to the regression performance. The results are consistent with the underlying biology: TFs and DNA methylation are the master regulators or upstream controls of the transcriptional program whereas miRNAs serve as a post-transcriptional fine-tuning rheostat [3]. Comparing with the findings in glioblastoma, however, where CNV played a major role in explaining gene expression [12], we suggest that the moderate effect of CNV observed here may be AML-specific, i.e., it is unlikely that CNV will have the same effect in other diseases. Indeed, recent studies have shown that many of the AML genomes lack structural abnormalities, implying that the disease complexity may likely reside at the transcriptional and epigenetic level [9].

**Table 1:** Model comparison.

	Spearman (%)	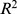 (%)	RACER vs X: p.value 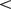	
RACER	60.0	31.0	Not applicable	
RACER, all excluding CNV	59.7	30.7	1.73E-01	4.59E-02
RACER, all excluding miRNA	59.6	30.5	7.41E-02	6.17E-03
RACER, all excluding DM	56.5	26.3	1.07E-44	4.29E-56
RACER, all excluding TF	43.0	17.8	3.42E-54	1.11E-53
RANDOM	0.18	0.00	1.17E-58	1.62E-58

RACER: full model; RACER, all excluding X: full model without using variable × 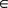 {CNV: copy number variation, miRNA: miRNA expression and seed match, DM: DNA methylation, TF: transcription factor binding signals}; RANDOM: full RACER on expression data with randomly shuffled gene symbols. “RACER vs X: p.value 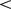
”: p-values indicate how significantly higher the Spearman and 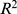 coefficients of the full RACER model, comparing with each reduced model based on Wilcoxon signed rank test. Spearman: Median Spearman correlation coefficients; 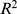: Median coefficient of determination.

The above correlation is based on the results after stage 1 regression (Eq 1). We also performed a similar model comparison using the predicted gene expression after stage 2 regression (Eq 2) and obtained consistent results (Figure S2; Table S1). Notably, however, the model performances at the two regression stages are not directly comparable due to the following subtleties. At stage 1, we trained a separate linear model 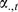 and performed 10-fold CV on **genes within each sample** 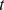. The model performance was then averaged over the 10 folds for each sample. As a result, each model had 173 Spearman correlations as data points, which were then plotted as boxplot in Figure S2A. In contrast, at stage 2 we trained a common set of linear coefficients 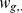 as the estimated regulatory relationships and performed 10-fold CV on **samples for each gene** 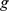. The model performance was then averaged over the 10 folds for each gene. As a result, each model had 16653 Spearman correlations (representing the 16653 genes) as data points (Figure S2B). Due to the much larger number of genes than the number of samples and the inherent sample-specific differences, the stage 2 regression is perhaps more challenging than the stage 1 regression task. As a result, we observed more performance fluctuation or increased variance at the stage 2 CV as shown in Figure S2B comparing with stage 1 performance (Figure S2A). Nonetheless, we would like to emphasize that the actual message we were trying to convey in Figure S2 is the *relative performance* gained by the full model comparing with the reduced models, which is remarkably consistent at both regression stages.

Finally, we further compared four alternative models each using copy number and DNA methylation data but different in using the remaining input data as follows:

1. TRANSFAC + TargetScan
2. TRANSFAC + TargetScan * miRNA.exprs
3. ENCODE + TargetScan
4. ENCODE + TargetScan * miRNA.exprs (the full RACER model)

Here, “TRANSFAC” represents the integer counts of the putative TF binding sites from TRANSFAC database (version 7.4) [23] corresponding to 282 TFs at the promoters of the 16653 target genes, “TargetScan” represents the putative miRNA binding sites from TargetScan database [21] at 3
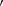
UTR of the target gene, and “TargetScan * miRNA.exprs” represents the target site counts weighted by the corresponding miRNA expression. Notably, model 1 is essentially the same as the model described by [12]. We then compared the four models in terms of the Spearman correlation between the predicted and observed mRNA target expression signals via 10-fold CV. Remarkably, we found that models (3) and (4) performed significantly better than models (1) and (2) (p 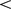 2.92E-53, Wilcoxon signed-rank test; Figure S3). In other words, the ENCODE TF binding data conferred significantly higher explanatory power than the TRANSFAC TF binding data for the mRNA expression level in the AML samples. One possible explanation would be that the *in vivo* ChIP-seq measurements in K562 are perhaps more consistent with the actual TF occupancies in the AML patient samples than the TF binding signals from the motif database. Although we observed no significant improvement by weighting the target site counts with the miRNA expression, we decided to still use the full RACER model (with miRNA expression) to more realistically recapitulate the regulatory relationships. Presumably, miRNAs with low or no expression (regardless of its potential cognate mRNA targets) should assume lower or no regulatory power than the highly expressed ones and vice versa.

### Power analysis of miRNA and TF target predictions

**Figure 3:**
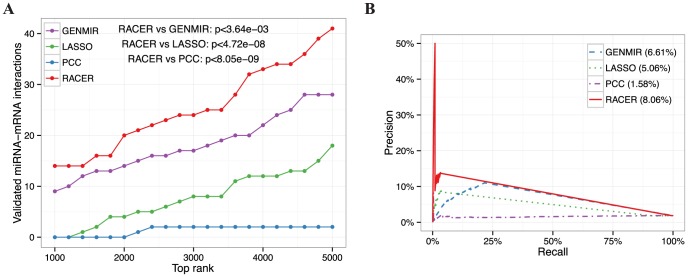
Power analysis of miRNA target predictions. We applied GenMIR++, LASSO, PCC, and RACER to score miRNA-mRNA interactions using the AML data. **A**. For each comparison method, miRNA-mRNA interactions were ranked based on their scores. The number of validated miRNA-mRNA pairs from MirTarBase [27] were then plotted as a function of the top rankings from 1000 to 5000 pairs with 200-interval. P-values indicate Wilcoxon signed rank test by comparing RACER with each of the three other methods. **B**. Precision-recall in detecting confidence targets of miR-34a in K562 obtained from a miR-34a transfection study coupled with mRNA pull-down or expression profiling [28]. Values in the parentheses besides each method indicate the corresponding areas under of the curve.

**Figure 4:**
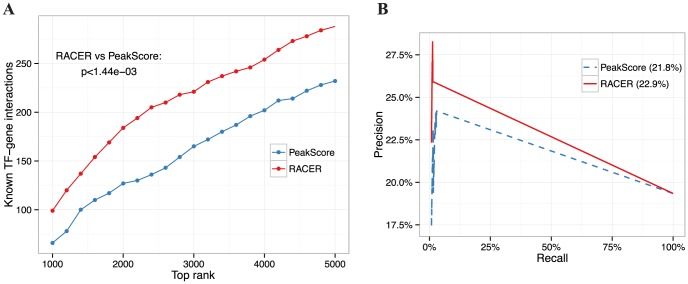
Power analysis of TF target predictions. **A**. Number of motif-based TF-gene interactions from TRANSFAC [23] as a function of top rankings from 1000 to 5000 interactions pairs with 200-interval. P-value indicates one-sided Wilcoxon rank-sum test by comparing RACER with PeakScore (i.e., ENCODE TF binding scores). **B**. Precision-recall of confidence targets of Gata2 in K562 obtained from a *GATA2* knockdown study [29]. Areas under of the curve are indicated in the parentheses.

We examined how well our model can be used to predict miRNA-mRNA and TF-gene regulatory relationships using 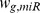 and 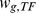 derived from Eq 2. For miRNA target predictions, we applied three other methods as comparison to predict miRNA targets using the same AML data, namely GenMiR++ (GENMIR; [24]), LASSO with only miRNA expression coupled with binary seed-match matrix as predictors [25], and Pearson correlation coefficient (PCC; [26]) (**Materials and Methods**). The evaluation of each method was based on the number of validated interactions it identified from MirTarBase [27] among the top 1000-5000 (with 200-interval) ranked prediction list, and the precision-recall in recovering the confidence targets derived from an independent miR-34a perturbation study [28]. For the latter, we constructed a panel of 338 putative positive target genes of miR-34a by intersecting the mRNA pull-down from biotinylated (Bi-)miR-34a and the down-regulated mRNAs upon miR-34a transfection in K562. Comparing with the other methods, RACER performed the best in identifying significantly more validated interactions among its top ranked predictions (p
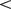
[1E-09, 1E-3], Wilcoxon signed rank test) (Figure 3A) and achieved the best precision-recall in miR-34a target prediction (Figure 3B). For TF-gene predictions, a similar test was conducted. Specifically, we compared the RACER predictions with peak scores 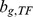 calculated by the ENCODE team [7]. We used as orthogonal data the motif-based interactions from TRANSFAC [23] and significantly down-regulated targets from a *GATA2* knockdown study in K562 cell line [29]. Comparing with using peak scores alone, the top rank list of TF-gene pairs from RACER are significantly more enriched for the motif-based interactions (Figure 4A), and the association scores 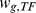 for GATA2-gene pairs exhibit higher precision-recall (Figure 4B). Together, RACER delivered competitive accuracy in predicting miRNA/TF-gene regulatory relationships. RACER's favourable performance is likely attributed to the linear decomposition of the various expression co-regulators, which may be over/underestimated in reduced or alternative model formalisms.

### Feature selection of predominant expression regulators in AML

To determine the most predominant TF/miRNA regulators in regulating gene expression in AML, we developed a feature selection procedure. Specifically, we performed the same regression analysis as described above but leaving out one of the TF/miRNA regulators, regulatorX, from the regression formula. We then compared the residual sum of squared (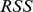) errors produced by the reduced RACER model denoted as 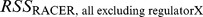 with the error from the full RACER model (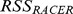) using 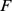-test, where the 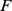-statistics is defined as:
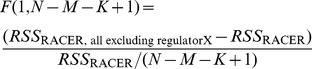
(3)where 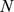, 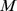, 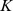 are the respective number of genes, miRNAs, and TFs; 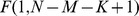 is the 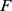 distribution with degree of freedoms (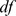): 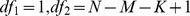. The *p*-value was then calculated by 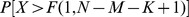 and adjusted for multiple testing over the 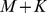 TF/miRNA regulators using Benjamini-Hocherberg method [30]. With false discover rate (FDR) 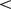 0.1, we identified 18 predominant transcriptional regulators consisting of 16 TFs and 2 miRNAs (Table S2). To examine the biological functions of these selected regulators, we performed a functional enrichment analysis using their associated targets having nonzero coefficients (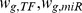) determined by RACER. Based on enrichments for biological processes from gene ontology (GO) database [31] and canonical pathways from MSigDB [32], we discovered several interesting functions related to the selected regulators (enrichment FDR
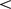
0.1; Table 2, S3). For instance, PHF8, Max, YY1 and C/EBP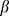 are engaged in regulating DNA repair whereas Maz and ELF1 are involved in tumor necrosis factor pathway.

**Table 2:** Selected regulators and the functional enrichments of the predicted targets by RACER.

Regulator	F-statistic	FDR	Enriched pathways or biological processes	Hits	Gene set	Enrichment FDR
PHF8	1565.63	0	misfolded or incompletely synthesized protein catabolic process (GO:0015693)	8	8	0
			DNA repair (GO:0006903)	77	168	1.18E-02
			REACTOME SIGNALING BY WNT	42	65	4.79E-06
			DNA repair (GO:0006903)	61	168	1.73E-04
Max	112.82	8.20E-24	REACTOME DNA REPAIR	44	112	6.91E-04
			KEGG BASE EXCISION REPAIR	17	35	6.06E-02
MAZ	64.14	2.34E-13	ST TUMOR NECROSIS FACTOR PATHWAY	15	29	4.82E-02
ZBTB7A	50.29	1.96E-10	REACTOME P38MAPK EVENTS	7	13	7.27E-02
PU1	31.50	2.30E-06	SA PTEN PATHWAY	6	17	3.11E-02
CCNT2	29.32	5.89E-06	REACTOME CDK MEDIATED PHOSPHORYLATION AND REMOVAL OF CDC6	22	48	4.96E-02
			REACTOME SIGNALING BY WNT	28	65	2.39E-02
hsa-miR-506	28.73	6.84E-06	REACTOME SYNTHESIS OF PC	4	18	2.03E-02
YY1	19.60	4.54E-04	DNA repair (GO:0006903)	50	168	1.92E-02
			REACTOME SIGNALING BY WNT	29	65	7.60E-05
CEBPB	14.11	6.53E-03	DNA repair (GO:0006903)	19	168	1.36E-02
			REACTOME P53 INDEPENDENT G1 S DNA	9	51	4.76E-02
			DAMAGE CHECKPOINT			
hsa-miR-548p	13.33	9.29E-03	ST ERK1 ERK2 MAPK PATHWAY	8	32	4.91E-03
			KEGG CHRONIC MYELOID LEUKEMIA	11	73	4.15E-02
ELF1	10.30	4.45E-02	ST TUMOR NECROSIS FACTOR PATHWAY	16	29	3.56E-02

To further examine the functional implication of the top regulators in AML, we performed an in-depth literature survey on AML. Remarkably, we found that many TF regulators among our top predictions have a role in leukemogenesis. To name a few (by the order of their statistical significance), the most significant regulator we found is PHF8, which is a histone demethylase that is specifically recruited by PML-RARa fusions. PHF8 enzymatic function is critical to the mechanism of all-trans retinoic acid treatment in acute promyelocytic leukemia [33]. The second most significant regulator Max interacts with c-Myc to form c-Myc/Max heterodimers, which regulate key genes involved in the proliferation and differentiation of hematopoietic cells. Disruption of the formation has been demonstrated to inhibit leukemic proliferation and induce apoptosis and differentiation [34], [35]. Furthermore, Max has been demonstrated as an important co-activator of C/EBP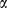 during granulocytic differentiation [36]. Maz (the 3rd significant regulator) is a proto-oncogene which regulates the expression of c-Myc; a study on 34 AML patients demonstrated prominent Maz expression level in 44% of primary patient samples versus only 8% of healthy donor samples [37], [38]. The fourth regulator ZBTB7A (LRF) is involved in several stages of hematopoiesis in both myeloid and lymphoid lineages and is highly expressed in both normal and malignant myeloid cells [39].

Moreover, PU1 was ranked at the fifth among the 18 regulators with FDR 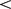 2.4E-06 and has been shown by Friedman (2007) to be one of the key regulators of early myeloid development in mouse model. In particular, mutant mice with reduced PU1 activities established via knockout experiments exhibited diminished monocytes and neutrophils [40]. The sixth regulator CCNT2 is a member of the p-TEFb complex that has been shown to inhibit differentiation of HL-60 and THP-1 cells. Down-regulation of CCNT2 by miR-29a and miR-142-3p resulted in induction of monocytic differentiation [41]. Further down our top regulator list, CHD1 (the tenth regulator) is a chromatin remodeling protein that is recruited to the proximal nucleosomes of actively transcribed genes, where it is responsible for nucleosome turnover and Pol II promoter escape [42]. In transformed mouse myeloid cells, CHD1 has been shown to be recruited to the promoter of *Hoxa9*, a Hox-containing transcription factor that is required for normal hematopoiesis and strongly stimulates myeloid cell proliferation [43]. The 11th regulator EGR-1 is a tumor suppressor gene located on q arm of the human chromosome 5. Deletion of the *EGR-1* locus is a recurrent genotype in patients with myelodysplastic syndrome and AML [44]. Additionally, haploinsufficiency of EGR-1 in combination with reduced TP53 activity has been shown to induce AML in mice [45].

Furthermore, C/EBP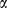 is another known myeloid developmental regulator [40]. Although we currently do not have ChIP-seq data for C/EBP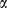, some literature suggested that C/EBP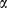 co-immunoprecipitates with endogenous C-Fos [46], which is the 14th regulator and the only prognostic marker revealed by our survival analysis below. Furthermore, C/EBP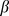 (the 15th regulator) has been shown to drive the differentiation of immature myeloid cells into granulocytes and is important for the retinoic acid-induced differentiation of acute promyelocytic leukemia cells [47]. Also, C-Myc is one of the known AML regulators listed by [48]. Based on our results, C-Myc is ranked at the 24th place among the regulators and has modestly significant statistics: 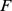-statistic 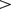 7.58; p-value 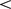 0.0059; FDR 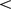 0.15. Despite previous suggestions by [46], we did not found p300 a significant regulator (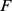-statistic 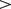 0.018; p-value 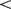 0.90; FDR 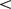 1), perhaps implying that p300 may have non-linear relationship with other TF/miRNA regulators, in which case it is not readily detectable via the 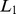-constrained linear regression approach. However, we did identify one of its interaction partners YY1, which is the 12th regulator in our list with FDR 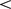 0.0005 (described in details below) [49].

For miRNA regulators, we first examined the statistics of those previously identified leukemia-related miRNAs, and found that they exhibited modestly significant statistics based on our analysis. For instance, previous studies showed that silencing of miR-145 in mouse hematopoietic stem/progenitor cells induced transition into a myeloid-like leukemia [50], [51]. In our analysis, miR-145 has p-value 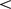 0.02 but did not pass multiple testing correction (FDR 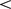 0.4). Additionally, miR-155 is another proposed AML-related miRNA [50], which was detected by our individual feature selection procedure but again did not survive the multiple testing correction (p
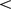
0.04; FDR
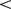
0.5). Presumably, the differences were due to experimental conditions and sample heterogeneity. Moreover, most of these miRNAs were primarily identified based on their individual effects in tumors rather than how well they perform to explain the underlying mRNA expression in a composite setting as demonstrated here.

To examine whether the miRNAs regulatory powers were overshadowed by the TF influence, we performed the same feature selection procedure without TF binding scores and compared the distributions of the 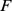-statistics for miRNAs in the full RACER (red) and the reduced RACER model (cyan) (Figure S4A). We found that excluding TFs in fact incurred an overall loss of explanatory power for the miRNAs, comparing with the full model (p
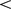
1.8E-4, Wilcoxon signed rank test). This is mainly attributable to a gain of explanatory power for the CNV and DNA methylation (DM) in the reduced model, in terms of both the magnitude of the linear coefficients (Figure S4B) and the 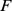-statistics (Figure S4C). Despite a general loss of explanatory power, however, miRNA miR-506 gained higher weights in the reduced model (cyan), comparing with the same miRNA in the full model (red) (Figure S4D). Additionally, we observed a more significant weight for miR-145 (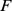-statistic 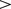
10.6, FDR
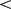
0.13) in the reduced model comparing with the same miRNA in the full model (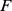-statistic 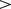
4.8, FDR
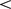
0.42). On the other hand, the weight of miR-155 has reduced from 4.2 to only 1.2.

Intriguingly, however, we identified hsa-miR-548p, a recently identified and poorly characterized miRNA (chr5:100,152,186-100,152,269 [-]; miRBase accession number: MI0006420; [52]). Notably, miR-548p is significant in both the full and reduced RACER model without TFs (FDR
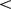
0.1; Figure S4D), indicating its robust explanatory power of AML mRNA expression. Importantly, the predicted target genes of miR-548p are enriched for Chronic Myeloid Leukemia (CML) (FDR
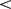
4.15E-02). Despite the distinct phenotypes between AML and CML in terms of the respective accumulation of immature and partially mature white blood cells, the underlying tumorigenic drivers are unclear. Remarkably, we discovered a direct interaction link inferred by RACER between miR-548p and Yin Yang 1 (YY1), which is itself one of the 18 selected regulators and a putative myeloid transforming gene [15] (discussed further in the network analysis below). Furthermore, [53] found that the MIR548p gene locus is located approximately in the middle of a copy number loss region (chr5:100,425,442-180,857,866) detected by aCGH (array comparative genomic hybridization) in a 39-year old male diagnosed with acute T-cell lymphoblastic leukemia, and no other known gene is located within 1 Mb vicinity outside of this genomic region. Accordingly, we propose an experimental investigation of the role of miR-548p in AML.

### Phenotypic implication of the 18 selected regulator activities in AML patients

**Figure 5:**
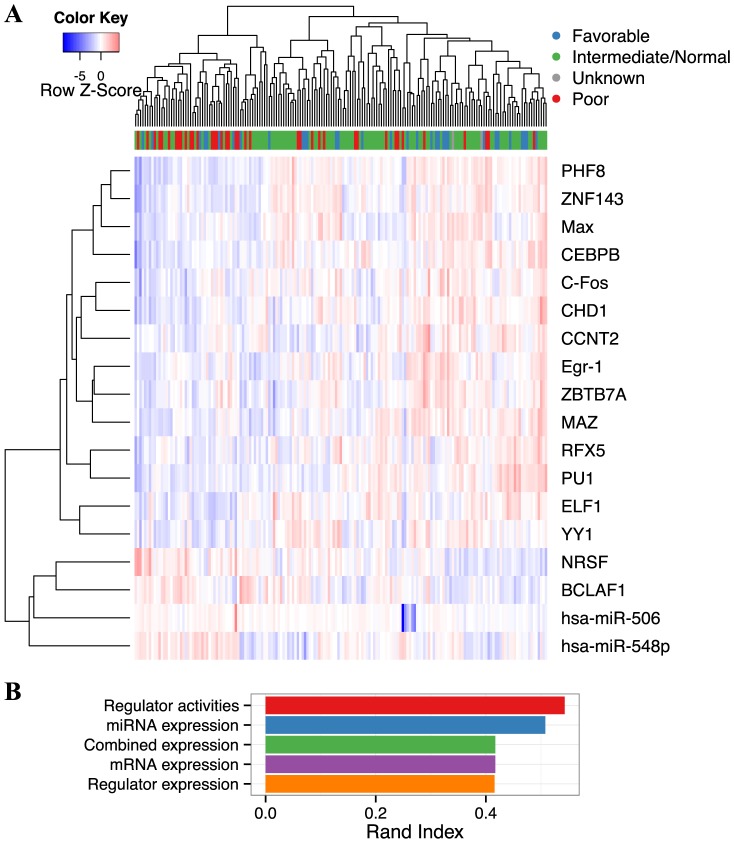
Selected regulator activities cluster cytogenetic risk groups. **A**. Heatmap of the inferred activities of the 18 selected regulators consisting of 16 TFs and 2 miRNAs. The bars under the dendrogram indicate four different cytogenetic risk groups [9] with colour legend indicated on the top right corner. **B**. Rand index of the clustering assignments using regulatory activities or expression profiles of either the selected regulators or all of the miRNAs/mRNAs. The higher the Rand index the more consistent the clustering pattern is to the cytogenetic group assignments.

We next examined the phenotypic implication of the above-identified 18 transcriptional regulators based on their inferred regulatory activities in the AML patient cohort. In particular, we examined how well the 18-regulator activity panel can cluster patients at different cytogenetic risks [9]. Remarkably, the clustering pattern of the regulatory activities is largely consistent with the risk groups (Figure 5A). Specifically, the cytogenetically poor group (red bar on the top of the heatmap) mostly cluster to the left major branch of the dendrogram and exhibit consistently lower activities (blue colour in the heatmap) for the top 14 regulators in comparison with cytogenetically favourable or normal group (blue or green bars). Quantitatively, clustering by the regulatory activities revealed higher consistency with cytogenetic risks than clustering by expression based on Rand index (Figure 5B; **Materials and Methods**).

**Figure 6:**
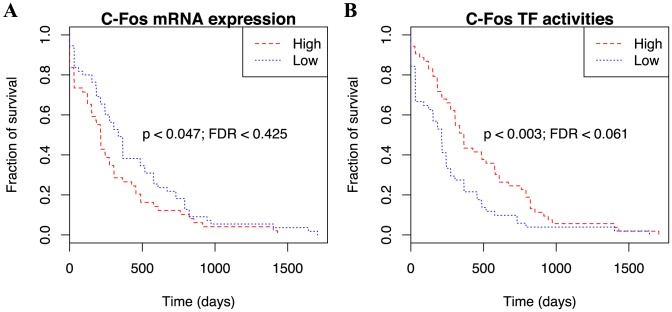
Kaplan-Meier survival analysis. AML patients with higher and lower than averaged C-Fos (**A**) mRNA expression or (**B**) inferred activities were divided into “High” (red dash) and “Low” (blue dot) groups, respectively. Survival fractions as a function of time (days) between initial diagnosis and death were then plotted for the two groups and the significant separation of the two curves were assessed by log-rank test followed by multiple testing correction over all of the 18 features to convert the resulting p-value to FDR.

Furthermore, we performed Kaplan-Meier survival analysis or log-rank test (**Materials and Methods**) using the 18-regulator activities in comparison with the same analysis using the corresponding mRNA expression profiles of the features. Based on the clinical data from TCGA, we characterized the level of associations of patient survival time from the date of diagnosis to death with whether their feature activities or mRNA expression are higher or lower than the sample average. After multiple testing correction, we obtained C-Fos as the only statistically significant prognostic marker among the 18 candidate markers. Importantly, while the mRNA expression level of C-Fos produced only modest separation between the two groups (p
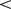
0.047; FDR
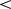
0.425; Figure 6A), the *activity* of C-Fos conferred a much more significant prognostic power (p
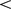
0.003; FDR
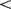
0.061; Figure 6B). Interestingly, while patients with high C-Fos mRNA expression exhibited poor survival outcome, the opposite was observed for patients with high C-Fos activities. Thus, the feature activities from our analysis may likely reveal cancer biology that is not readily observed by expression analysis alone.

### Topology of AML regulatory network involving the selected regulators

**Figure 7:**
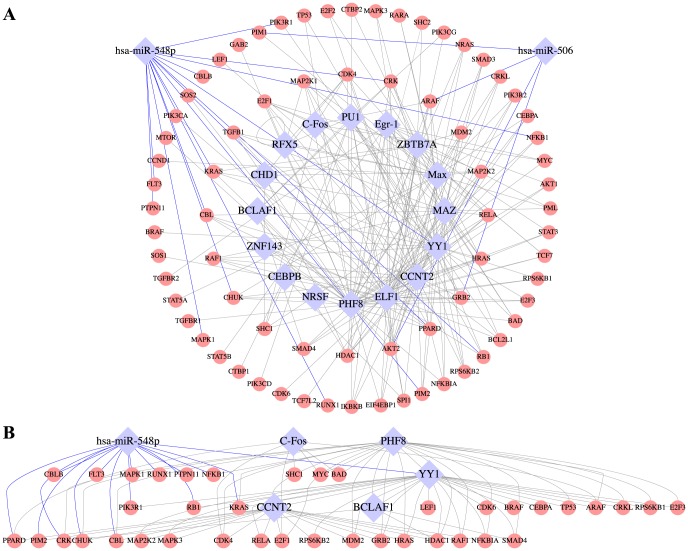
AML regulatory network. **A**. The network drawn by Cytoscape 3 [54] comprises 18 selected regulators (blue diamond) having nonzero putative interactions with leukemia-related genes (red circle) obtained from COSMIC or MSigDB. The blue and grey edges indicate miRNA-mRNA and TF-gene regulatory relationships, respectively. **B**. A subnetwork containing the selected regulators and their targets. The selected regulators form a 3-layer hierarchical structure with 3 master regulators hsa-miR-548p, C-Fos, PHF8 on the top layer, one intermediate regulator YY1 in the middle layer, and two downstream regulators CCNT2 and BCLAF1 arranged at the bottom layer.

To visualize in network context the regulatory relationships between the 18 selected regulators and their predicted leukemia-related target genes, we imported into Cytoscape [54] the TF-gene and miRNA-mRNA pairs involving nonzero connectivity between the 18 regulators and the leukemia genes filtered by leukemia-related pathways from MSigDB [32] and leukemia-related genes from COSMIC [55]. In Figure 7, the selected regulators and target genes are displayed in blue diamond and red circle, respectively. Connections between miRNA-mRNA (TF-gene) are coloured in blue (grey). The resulting regulatory network is densely connected, implying that the regulators we identified shared many common leukemia-related target genes. The select subnetwork assumes a hierarchal structure (Figure 7B) reminiscent of the 3-layer network architecture suggested by [10]. Specifically, hsa-miR-548p, C-Fos, and PHF8 form the top layer (master regulators), YY1 the middle layer (regulators that is regulated by the master regulators above it and regulate other TFs below it), and CCNT2 and BCLAF1 the bottom layer (regulators that are regulated by the middle layer and only regulate non-TF targets). Remarkably, the fact that miR-548p regulates YY1 and a large cohort of AML-related genes either directly via base-pairing or indirectly via YY1 provides a further support of its important role as a master regulator in AML. Indeed, the role of YY1 in hematologic malignancies has been implicated in various cancers including AML [56], in which ectopic YY1 expression can contribute to AML malignancy by interfering with the normal myeloid differentiation program [15]. Moreover, YY1 has been reported to indirectly engage in histone modification by recruiting histone deacetylases HDAC1-3 and histone acetyltransferases such as p300 to diverse number of promoters in order to respectively activate or repress the promoters [49]. Therefore, an immediately appealing follow-up study from our current analysis would be to experimentally validate the interaction between the two key expression regulators hsa-miR-548p and YY1 in AML patient samples or cancer cell-lines such as K562.

## Discussion

In this study, we demonstrated via a simple two-stage regression framework that mRNA expression level in AML can be best explained by integrating various genome-wide measurements including CNV, DNA methylation, miRNA expression from TCGA [9] coupled with sequence-based miRNA-mRNA interactions from TargeScan [21], and TF binding data from ENCODE [7]. The proposed corresponding regression model namely RACER enabled us to infer the activities and associated target genes of the AML-specific expression regulators. Remarkably, the identified regulator activity patterns were highly consistent with the cytogenetic profiles and exhibited promising prognostic power, comparing with using expression profiles alone.

Besides the model refinement upon the previous related works [12], [13], the success of our model may also be attributed to the lack of DNA mutations in the AML patients [9], which if were prevalent might have confounded our model to some extent. In particular, although CNV was incorporated into RACER, we did not scrutinize the TF/miRNA regulation changes due to single nucleotide polymorphisms (SNPs) or indel mutations. Additionally, we used the TF binding profiles measured in the generic cell-line namely K562 from ENCODE as surrogates for each AML sample. While we observed a good expression correlation between the averaged AML samples and K562 expression processed by ENCODE (Figure S5), the results may differ if the variance of the actual TF occupancy across AML samples is high. Also, the aberrant miRNA activities may induce changes in the mRNA translation in AML, which can be detected by fitting our model on protein output (which is currently unavailable) as response variable. Lastly, we assumed a linear combination of the regulator effects on mRNA level. A different model formalism is of interest in future work to account for potential synergistic or non-linear regulatory relationships that has been suggested in previous studies [57].

Also, the ChIP-seq data from ENCODE were available for only a subset of the TFs in a restricted number of cell-lines. For instance, the genome-wide occupancy data of some of the AML-related TF regulators such as C/EBP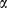, STAT1-3, WT-1, c-Myb as listed by [48] are not yet available in the (untreated) K562 from the ENCODE Phase II project. Perhaps a reasonable alternative would be using the TRANSFAC data, which has been demonstrated by [12], [58], and others. Notably, however, the main novelty of RACER lies its ability to successfully integrate the ENCODE and TCGA data, and we observed that ENCODE TF binding data conferred a significantly higher explanatory power than the TRANSFAC data in explaining the mRNA expression of the AML patient samples (Figure S3). Nonetheless, one could easily switch the TF binding matrix from ChIP-seq signals to other pre-calculated TF binding sites as demonstrated in the model comparison above.

During the preparation of this manuscript, [58] published a similar model called ISMARA (Integrated System for Motif Activity Response Analysis), which uses Bayesian regression with Gaussian prior distribution to infer the *motif activities*. The model takes as input the motif counts at the promoters or 3
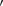
UTRs and predicts the promoter expression (i.e., without using CNV, DNA methylation, ChIP-seq signals, and miRNA expression as in RACER). In terms of target predictions, ISMARA was designed to accurately predict regulatory relationships between *motifs* and *promoters* rather than the exact TF/miRNA-target interactions. On top of that, ISMARA provides a very nice web interface (https://ismara.unibas.ch/fcgi/mara) highly accessible to non-computational researchers. While the LASSO approach minimizes the squared error + *L*_1_-norm to obtain *point estimates* of the regulatory activities, the Bayesian model in ISMARA infers the *posterior distribution* of the regulator activities by combining the Gaussian likelihood with the Gaussian priors for the activities. Since we do not have a gold-standard for the true regulatory activities in AML, it is difficult to directly compare the performance between ISMARA and RACER. As mentioned by Balwierz *et. al.* (2014), however, the LASSO approaches can be interpreted as a Bayesian model using Laplacian priors instead of Gaussian priors in the regression framework and remain as a popular alternative since they induce a sparse solution (i.e., irrelevant parameters will be set strictly to zero after the fitting) in the high dimensional feature space [58].

Importantly, our regression analysis coupled with feature selection provided two novel biological findings involving a recently discovered miRNA hsa-miR-548p and a TF regulator C-Fos. Specifically, we observed that miR-548p not only conferred significant explanatory power to mRNA expression changes in AML but also targets a large number of leukemia-related genes. Moreover, our network analysis identified miR-548p as a master regulator that regulates another selected regulator and also a prominent AML-related TF YY1 [15], which is known to have a broad regulatory spectrum due to its association with histone modifiers such as HDACs and p300 [49]. In addition, our analysis suggested C-Fos as a novel prognostic marker, whose inferred regulatory activities across the AML samples are more coherent with patient survival rate than its corresponding mRNA expression.

While C-Fos exhibited ectopic expression in various clinical tumour tissues, the predictive power of C-Fos mRNA expression as prognostic marker is often inconsistent among different studies [16]. Although a validation dataset that can be used to directly address our question is currently unavailable, we conducted an in-depth literature survey and found several lines of evidence pertinent to the proposed prognostic value of C-Fos in AML. C-Fos is traditionally viewed as an oncogene that is highly expressed in many solid tissue cancers and serves as an excellent marker for cancer progression and negative prognosis (reviewed in [59]). The role of C-Fos as a prognostic marker in AML has only been investigated in relatively small cohorts and remains largely unclear. Previous work on the expression profiling of untreated and relapse AML in 18 patients showed a significant increase of C-Fos mRNA at relapse [60]. Subsequent studies aimed at defining prognostic markers of AML, however, did not identify a significant association between C-Fos expression and survival [61], [62]. In contrast, a study on the effects of a histone deacetylase inhibitor, Vorinostat, in cell lines and AML patient-derived cells demonstrated an increase in C-Fos mRNA expression following treatment in conjunction with an increase in apoptosis and differentiation [63]. Furthermore, several recent studies have raised the idea that C-Fos may have tumor-suppressor activity [64]–[67]. For instance, [66] and [67] showed that the loss of C-Fos expression is associated with tumour progression in ovarian and gastric carcinoma, respectively. Indeed, our results show that patients with higher C-Fos activities correspond to poor prognosis (Figure 6). While the exact mechanism by which C-Fos contributes to tumor suppression is unclear, it is possible that C-Fos mediates apoptosis through the p38 MAP kinase pathway [68] or via induction of Fas ligand as observed in a human T-cell leukaemia cell line [69]. With more data becoming available, we look forward to further validating our novel finding of C-Fos in terms of its prognostic value in AML.

Although our analyses identified several AML-related TFs such as PU1, C-Fos, YY1, some of the other AML-related TF regulators such as p300 and C-Myc [48] exhibit either no or rather modest significance (e.g., 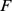-statistic 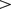
7.58; p-value 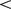
0.0059; FDR
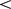
0.15 for C-Myc) based on our analysis, which may indicate an over-stringent cutoff (FDR
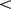
0.1) used in the feature selection procedure or the intrinsic difference between the mRNA expression and the cognate TF activities. The differences may be also due to various experimental conditions and sample heterogeneity. Also, some of those AML-related regulators were primarily identified based on their *individual* effects (e.g., via individual perturbation experiments in mice [40]) rather than how well they perform to explain the underlying mRNA expression in a *composite* setting as manifested here in the form of multivariate linear regression model. Presumably, TF activities is a function of its DNA binding efficacy, its interactions with Pol II, interplays with other trans-acting factors such as miRNAs or chromatin modifiers, and its catalytic abilities for promoting transcription, which may intrinsically vary due to (for instance) post-translational modification despite its low expression variance across AML patients. Notably, we can infer the regulatory activities of the proposed regulators in a single sample without borrowing information across multiple samples, which is cost-effective for future screening of unknown cases in a prognostic setting. In conclusion, our proposed approach provides a novel strategy and, to the best of our knowledge, the first framework that successfully integrates TCGA and ENCODE data to study AML at a comprehensive system level.

## Materials and Methods

### Collection and preprocessing of the AML data from TCGA

Genome-wide measurements including mRNA/miRNA expression profiles, DNA methylation (DM), and copy number variation (CNV) and clinical information for Acute Myeloid Leukemia (AML) were downloaded from TCGA Data Portal [9]. The AML data contain 173 samples with measurements available across all four platforms. For all of the data, we used the Level 3 processed data. For expression data, processed RNA-seq and miRNA-seq data were used, which record the RPKM (read per kilobase of exon per million mapped reads) values for mRNA and RPM (reads per million miRNA mapped) for miRNA. The data were further log2-transformed and mean-centred. For CNV, we used the genome-wide SNP array data and the “Segment_Mean” scores. We first intersected the genomic coordinates (hg19) of the probes with the exon coordinates of the RefSeq genes. For genes having multiple scores, we took the average of the scores. For DM data, we used the Human Methylation 450 array data and “Beta_value” as the methylation scores. Multiple scores for the same gene were averaged.

### Preprocessing transcription factor binding data from ENCODE

The TF-binding data were retrieved from the ENCODE data portal [14]. All available TFs for K562 cell lines flagged as “treatment = None” were retrieved in narrowPeak format. In the case of duplicate items, tracks were chosen based on their labs, and priority was given to those done by labs contributing more samples. Tracks were named according to the antibodies used to immunoprecipitate the TF. In total, we obtained TF binding profiles for 97 TFs. Similar to the definition by [11], we defined the promoter region for each gene as their transcription start site (TSS) +/−50 bp based on Gencode V7 annotation [70]. We then intersected the list of promoters regions with the ChIP-seq coordinates for each TF and averaged the TF binding scores for multiple peaks to represent a single binding site per TF-gene pair. As a result, we obtained an 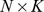 TF binding profile matrix between 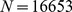 and 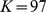 distinct genes (that have expression measurements in TCGA) and TFs, respectively.

### miRNA target site information

For each mRNA-miRNA pair that has measured expression in the TCGA data, we obtained their conserved target sites from TargetScanHuman 6.2 database [21]. For multiple target transcripts of the same gene, we used the transcript with the longest 3
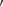
UTR [71]. This resulted in an 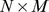 target site count matrix 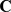 between 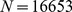 and 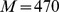 distinct genes and miRNAs.

### Power analysis of miRNA/TF-gene interactions

For miRNA target predictions, experimentally validated miRNA-mRNA pairs were downloaded from MirTarBase [27]. To compare the performance of RACER in predicting validated miRNA-mRNA interactions, we chose three existing methods namely Pearson correlation coefficient (PCC) [26], GenMiR++ [24], LASSO [25]. Specifically, PCC between each pair of m/miRNA across 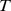 samples was computed using R built-in function cor. GenMiR++ was ran in Matlab with default setting using as input the binary target site matrix and the expression profiles. We implemented LASSO using *glmnet* with 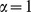 except that the best 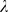 was chosen using cross-validation function cv.glmnet [72]. Specifically, we used expression of the miRNAs that have nonzero target sites to model the corresponding mRNA expression: 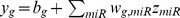. Due to the lack of validated targets, conventional power analysis such as ROC (Receiver Operating Characteristic) cannot distinguish the method performances. Instead, we assessed each method by the number of validated targets in their top ranked 1000 to 5000 targets with 200-interval.

In addition, we constructed a confidence positive target list for miR-34a using the published data from [28]. Specifically, we defined the positive hits as the intersect between the mRNA pull-down from biotinylated (Bi-)miR-34a and down-regulated mRNA upon miR-34a transfection in K562. For each method, we assessed its precision and recall (PR) using their prediction scores and summarized the performances by the area under the PR curve (AUC). For a given score cutoff, the precision and recall are estimated as the respective ratios of TP/(TP+FP) and TP/P, where TP and FP are the numbers of true and false positives, and P are the total number of positive miR-34a targets in the test data. The statistics were obtained using *ROCR* package [73].

For TF target predictions, we used the motif-based TF-target relationships from TRANSFAC (version 7.4) [23]. Similar to the above miRNA analysis, we assessed the detection power of RACER and peak scores from ENCODE in identifying the TRANSFAC interactions among the 1000∶200∶5000 ranks. Moreover, we constructed a confidence target gene list from genes with significant expression fold-change due to *GATA2* knockdown in K562 using the published data from [29] (i.e., processed table from Gene Expression Omnibus (GEO) with accession GSM798059). The same PR analysis was performed as described above.

### GO terms and pathways used in the functional enrichment analysis

We examined whether the predicted miRNA/TF targets of the selected regulators are biologically meaningful via functional enrichment analysis. Specifically, we downloaded the Gene Ontology (GO) terms in Biological Processes (BP) (GO-BP) using getBM function from R package *biomaRt* [74]. We filtered out GO terms with fewer than 5 genes or with evidence codes equal to Electronic Annotation (IEA), Non-traceable Author Statement (NAS) or No biological Data available (ND), which yielded 2007 GO-BP terms and 10315 unique genes [75]. Additionally, we downloaded canonical pathways from MSigDB (i.e., c2.cp.v4.0.symbols.gmt [32]). For each target list, we assessed their enrichment for each GO-BP term or pathway by hypergeometric test using R built-in function phyper [75]. The resulting p-values were adjusted for multiple testings with the BH-method over all of the GO terms or pathways by R function p.adjust to produce false discovery rates (FDR).

### AML phenotypic analysis

Clinical data for each of the 173 patients were obtained from TCGA data portal. We first performed a hierarchical clustering (hclust in R) using the feature activities or mi/mRNA expression profiles. In all cases, the distance and clustering method were set to 1 - Pearson correlation and average-linkage, respectively. We then examined how well each molecular signature can cluster the four cytogenetic risk groups. Specifically, we cut the dendrogram of each clustering assignments into 4 groups using cutree function from R and compared the resulting groups with cytogenetic assignments quantitatively using Rand index (RRand in R package *ClassDiscovery*). Additionally, we performed Kaplan-Meier survival analysis using R package survival to examine the prognostic power of the identified feature using either their inferred activities or mi/mRNA expression level. The resulting p-values from log-rank test were then adjusted for multiple testings with BH-method [30].

### RACER implementation and availability

RACER was implemented in R. Each of the two regression steps (Eq 1,2) was performed using *glmnet* with 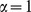 except that the best 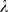 was chosen using cross-validation function cv.glmnet [72]. The source code together with the data used in this study are available at www.cs.utoronto.ca/~yueli/racer.html.

## Supporting Information

Figure S1 **Model comparison.** Boxplot of coefficients of determination between the predicted and the actual held-out genes in 10-fold cross-validation (CV). Please refer to Figure 2 legend in the main text for more details.
(EPS)

Figure S2 **Model comparison in regression step 1 and 2.** **A**. In regression step 1, correlation was calculated between predicted and observed gene expression within each sample by training on 90% of the genes and testing on 10% of the remaining genes in a 10-fold cross-validation (CV). **B**. In step 2 regression, we applied a similar 10-fold CV for each gene by training on 90% of the samples and testing on the remaining 10%. For each gene, we averaged Spearman correlation over the 10 runs, and compared the distributions of the correlation among the full, reduced, and random models. Please refer to Figure 2 legend for more details.
(EPS)

Figure S3 **Comparison of four alternative models.** Each model used copy number and DNA methylation data but different in using the remaining input data as indicated in the figure legend above. We compared the four models in terms of the Spearman correlation between the predicted and observed mRNA target expression signals via 10-fold cross-validation. The indicated p-value was derived from the comparison between the ENCODE-based models and the TRANSFAC-based models using Wilcoxon signed rank test.
(EPS)

Figure S4 **Comparison of miRNA explanatory powers in the full (red) and reduced RACER model excluding TF effects (cyan).** **A**. Comparison of *F*-statistic distributions of the miRNAs derived from the feature selection procedure applied to the full data (red) and the data excluding TF binding scores. **B** & **C**. Comparison of the activities and *F*-statistic of CNV and DNA methylation (DM), respectively. **D**. *F*-statistics of selected miRNAs for the full (red) and reduced RACER model with TF effects excluded from the linear equation (cyan). False discover rates (FDR) corresponding to the adjusted *p*-value of the *F*-statistic were indicated on the top of each bar.
(EPS)

Figure S5
**Expression correlation between AML averaged sample and K562 from ENCODE.** To compare the expression between AML samples and K562 (cell line derived from a CML patient), we obtained the mRNA expression (in RPKM based on Gencode V7) in K562 from the ENCODE Data Portal. Only expression in cytosol were used. Expression in replicates were averaged and logarithmically transformed with 0.05 as pseudo-count to stabilize variance (i.e., 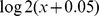). For the AML mRNA expression data, we averaged the mRNA expression across the 173 samples. The expression profiles between AML and K562 are significantly correlated (Pearson correlation 0.77; p
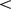
2.2E-16, correlation test via R function cor.test).(EPS)

Table S1 **Summary statistics in regression step 1 and 2.**
(PDF)

Table S2 **Feature selection results for the TF/miRNA regulators.**
(XLSX)

Table S3 **Functional enrichments of predicted targets from the 18 selected regulators.**
(XLSX)
