# Supplementary material for: Regression Analysis of Combined Gene Expression Regulation in Acute Myeloid Leukemia
Source: PLoS Comput Biol. 2014 Oct 23;10(10):e1003908. doi: 10.1371/journal.pcbi.1003908 (PMC4207489; doi:10.1371/journal.pcbi.1003908)
Supplement: Table S1 — Summary statistics in regression step 1 and 2. (PDF) [file pcbi.1003908.s006.pdf]

Table S1: Summary statistics in regression step 1 and 2

	Spearman at stage 1 & 2		RACER vs X: p.value <	
RACER	6.00E-01	4.63E-01	not applicable	
RACER, all excluding CNV	5.97E-01	4.59E-01	1.73E-01	7.75E-03
RACER, all excluding miRNA	5.96E-01	4.50E-01	7.41E-02	3.13E-08
RACER, all excluding DM	5.65E-01	4.33E-01	1.07E-44	2.54E-16
RACER, all excluding TF	4.30E-01	1.54E-01	3.42E-54	0.00E+00
Random	-1.83E-03	-1.65E-03	1.17E-58	0.00E+00

RACER: full model; RACER, all excluding X: full model without using variable X ∈ {CNV: copy number variation, miRNA: miRNA expression and seed match, DM: DNA methylation, TF: transcription factor binding signals}; RANDOM: full RACER on expression data with randomly shuffled gene symbols. Spearman at stage 1 & 2: Median Spearman correlation coefficients at regression stage 1 (Eq 1) and 2 (Eq 2) for each model, respectively. "RACER vs X: p.value <": p-values indicate how significantly higher the Spearman of the full RACER model comparing with each reduced model based on one-sided Wilcoxon rank-sum test at regression step 1 and 2, respectively.
